# Antibiotics’ consumption to early detect epidemics of P. aeruginosa in a burn center: a paradigm shift in the epidemiological surveillance of nosocomial infections

**DOI:** 10.1186/2047-2994-4-S1-P232

**Published:** 2015-06-16

**Authors:** A Fournier, P Eggimann, O Pantet, M Krähenbühl, C-L Bonnemain, C Fournier, J-L Pagani, J-P Revelly, E Dupuis-Lozeron, F Sadeghipour, A Pannatier, P Voirol, Y-A Que

**Affiliations:** 1 Pharmacy, CHUV, Lausanne, Switzerland; 2 Critical Care, CHUV, Lausanne, Switzerland; 3 Faculty of Biology and Medicine, CHUV, Lausanne, Switzerland; 4 Research Center for Statistics, University of Geneva, Geneva, Switzerland

## Introduction

The control of antibiotic resistance and nosocomial infections are among the major challenges for specialized burn centers. Early detection of those epidemic outbreaks is crucial to limit the human and financial burden.

## Objectives

We hypothesize that data collected in the frame of antibiotic consumption medico-economic surveys could be used as warning signal to detect early nosocomial outbreaks.

## Methods

Retrospective analysis including all burn patients staying more than 48 h and receiving systemic therapeutic antibiotics admitted to the Lausanne BICU between January 2001 and October 2012. Infection episodes were characterized according to predefined criteria. Antibiotic consumption data, obtained from the quarterly surveillance of drug consumption surveys, were translated in defined daily doses (DDDs).

## Results

297 out of 414 burn patients stayed more than 48 h for a total number of 7458 burn-days. We identified 610 infection episodes (burn wound [32.0%], respiratory [31.1%], catheter [21.8%]), due to 774 microorganisms. *Pseudomonas aeruginosa* (26.2%), *Staphylococcus aureus* (11.5%), *Candida albicans* (7.0%) were the main pathogens. We observed three distinct outbreaks of *P. aeruginosa* infections (2002-2003, 2006 and 2009-2011). These outbreaks were correlated with an increase in the DDD of anti-*Pseudomonas* antibiotics.

## Conclusion

Our data support **a paradigm shift in the epidemiological surveillance** of nosocomial *P. aeruginosa* epidemics in Burn Centers, by using the rise in antibiotic consumption as an early trigger to initiate molecular typing of *P. aeruginosa* strains and reinforcement of standard infection control procedures.

## Disclosure of interest

None declared.

